# Louisiana State Policies Prove Problematic for Pharmacist–Physician Collaboration

**DOI:** 10.5888/pcd17.200051

**Published:** 2020-08-20

**Authors:** Anna L. Hamilton, Marie Darr

**Affiliations:** 1 Well-Ahead Louisiana, Louisiana Department of Health, Baton Rouge, Louisiana

In a nation that spends more than 17% of its gross domestic product on health care, where health care costs are rising faster than costs in any other industry, one of our roles as public health practitioners is to identify and promote efficient care-delivery models (1). Well-Ahead Louisiana, a chronic disease prevention and health care access initiative of the Louisiana Department of Health, helps health care facilities identify and implement strategies to optimize efficient care. Well-Ahead Louisiana has partnered with pharmacists to increase the use of collaborative drug therapy management (CDTM) agreements, a proven method for improving patient outcomes by maximizing the pharmacist’s ability to practice at the top of their license in a team-based setting (2). True collaboration between clinicians and pharmacists, leveraging the unique expertise of both providers, has demonstrated significant improvements in patient outcomes (3,4). However, we encountered legal restrictions that prohibited small, rural pharmacies in Louisiana from establishing such agreements. Rural regions of Louisiana are more likely than nonrural regions to be designated as primary care health provider shortage areas (HPSAs) and could benefit most from the improved provider coordination that CDTM provides. We believe altering these regulations will make CDTMs a viable tool for pharmacists in rural Louisiana.

CDTM agreements, also known as collaborative practice agreements (CPAs), are legal accords between a pharmacist and a provider that allow the pharmacist to assume increased responsibility over patient care functions. First initiated in Washington State in 1979, CDTM agreements permit pharmacists, without the direct approval of a physician, to initiate, modify, or discontinue drug therapy, order and interpret laboratory tests, and advise patients on control of chronic conditions. Currently, 48 states and the District of Columbia have some form of authorized CDTM (5). The efficiencies created by CDTM have increased access to care, facilitated patient care management, and improved chronic disease outcomes, such as blood pressure control and hemoglobin A_1c_ regulation among people with diabetes (2).

In July 2018, Well-Ahead Louisiana launched an initiative to increase use of CDTM to treat heart disease and diabetes in rural areas. Because Louisiana has the fourth-highest diabetes prevalence and fifth-highest heart disease prevalence in the nation, our team targeted 4 geographic regions of the state, primarily rural, where the burden of these diseases was greatest (6). To assess pharmacists’ use of and familiarity with CDTM, Well-Ahead Louisiana fielded a SurveyMonkey questionnaire to 55 community pharmacies, 15 of which responded, and also conducted a short telephone interview with similar questions among 7 hospital pharmacies. Of the 22 respondents, only 1 pharmacist reported participating in an active CDTM. Answers ranged from “familiar” to “no experience” with CDTM, and several noted a lack of knowledge of the requirements and benefits of CDTM among pharmacy staff members and leadership. We also contacted the Louisiana Board of Pharmacy and the Louisiana State Board of Medical Examiners, reviewed CDTM policies in other states, and reviewed available online resources in Louisiana. According to Louisiana Board of Pharmacy records, only 75 (<1%) of the 9,087 licensed pharmacists in Louisiana participate in an active CDTM. After the interviews, Well-Ahead selected 2 rural hospital pharmacies and 1 community pharmacy to pursue the establishment of a CDTM agreement based on readiness and location in Well-Ahead Louisiana’s priority regions. 

Well-Ahead Louisiana has developed Louisiana-specific tools for CDTM and made them available to providers and pharmacists on its website (www.walpen.org/mtm). These tools include provider outreach guides, an overview of Louisiana regulations and requirements, and worksheets to assess readiness and capacity. In providing this technical assistance, Well-Ahead identified several barriers to establishing CDTM agreements:

- Agreements in Louisiana limit pharmacists to treatment of anticoagulation, diabetes, asthma, and dyslipidemia; smoking cessation; and providing vaccinations unless approved by the Louisiana State Board of Medical Examiners, despite evidence demonstrating CDTM benefits for other purposes, such as hormonal contraception, hepatitis C treatment, and HIV treatment (2).
- Louisiana is among 22 states that allows only a physician to collaborate (7), restricting CDTM agreements to a collaboration between a pharmacist and physician only. Of the 48 states that allow CDTM agreements, 23 allow any prescriber to enter into the agreement, and 3 allow any physician or nurse practitioner.
- The collaborating physician must be “physically present daily” to enter into such an agreement (8). Louisiana is 1 of only 3 states with such a proximity requirement; a fourth state requires the physician and pharmacist be located in the same practice (7).
- The paperwork associated with a CDTM is extensive. In contrast to less burdensome requirements in several other states (7), Louisiana requires documentation of demographic characteristics, the condition to be managed, drug substitutions, and the type and extent of drug therapy management for each patient. Detailed follow-up documentation of all physician consultations with the pharmacist and monthly patient status reports must be available in the event of an inspection. Adding to the administrative burden, agreements must be renewed annually and approved by the Louisiana State Board of Medical Examiners (9).

As we delved into the effect of these stipulations, we found that they rendered CDTM agreements impractical for many providers.

One of our partner sites, an independently owned community pharmacy, was interested in collaborating with a nearby rural health clinic. Both providers were eager to use a CDTM agreement as a tool to support their collaborative management of a subset of patients with diabetes. However, as with many rural providers, the clinic was managed by a nurse practitioner under another collaborative practice agreement with a physician who was only occasionally present onsite. Clarification from the Louisiana State Board of Medical Examiners indicated that the physician could not participate in CDTM because the physician was not physically present at the clinic at all times. The nurse practitioner is not considered an eligible provider for a CDTM agreement in Louisiana. Therefore, the agreement could not be pursued.

Another of our partner sites, a rural hospital pharmacy, planned to provide medication therapy management (MTM) services to patients referred by a nearby heart specialty clinic. Both parties were interested in pursuing a CDTM agreement as a tool to strengthen the benefit of the MTM visit. However, patients selected by the heart specialty clinic for MTM were often referred to the hospital pharmacist within 24 hours of seeing the physician and then seen by the pharmacist within 48 hours. This created only a 3-day window for a unique order set to be documented, including patient consent. Both parties preferred to continue using traditional authorization pathways rather than hurriedly submitting the paperwork required for CDTM.

On the basis of our experience with our 3 partner pharmacies, in addition to feedback from the 15 community pharmacies and 7 hospital pharmacies surveyed, we believe several changes to the current CDTM rules could increase use of CDTM and improve patient outcomes in Louisiana. We know of no historical barriers in Louisiana that would prevent consideration of these changes.

- In consultation with specialist stakeholders, consider adding hormonal contraception, hepatitis C, and HIV to the conditions and diseases eligible for CDTM agreements.
- Expand the definition of providers eligible to participate in a CDTM agreement to include nurse practitioners and other advanced-practice nurses. This expansion would increase the number of primary care sites that can participate, particularly in rural areas. Louisiana ranks 30th nationally in the number of primary care physicians per 100,000 residents (10), and more than 84% of the state’s land area is designated as a health professional shortage area by the Health Resources and Services Administration (HRSA) (Figure). It is of critical importance to the health of rural residents that providers and pharmacists in these underserved areas have opportunities to maximize their collaboration, thereby expanding treatment options for those residents.

**Figure:**
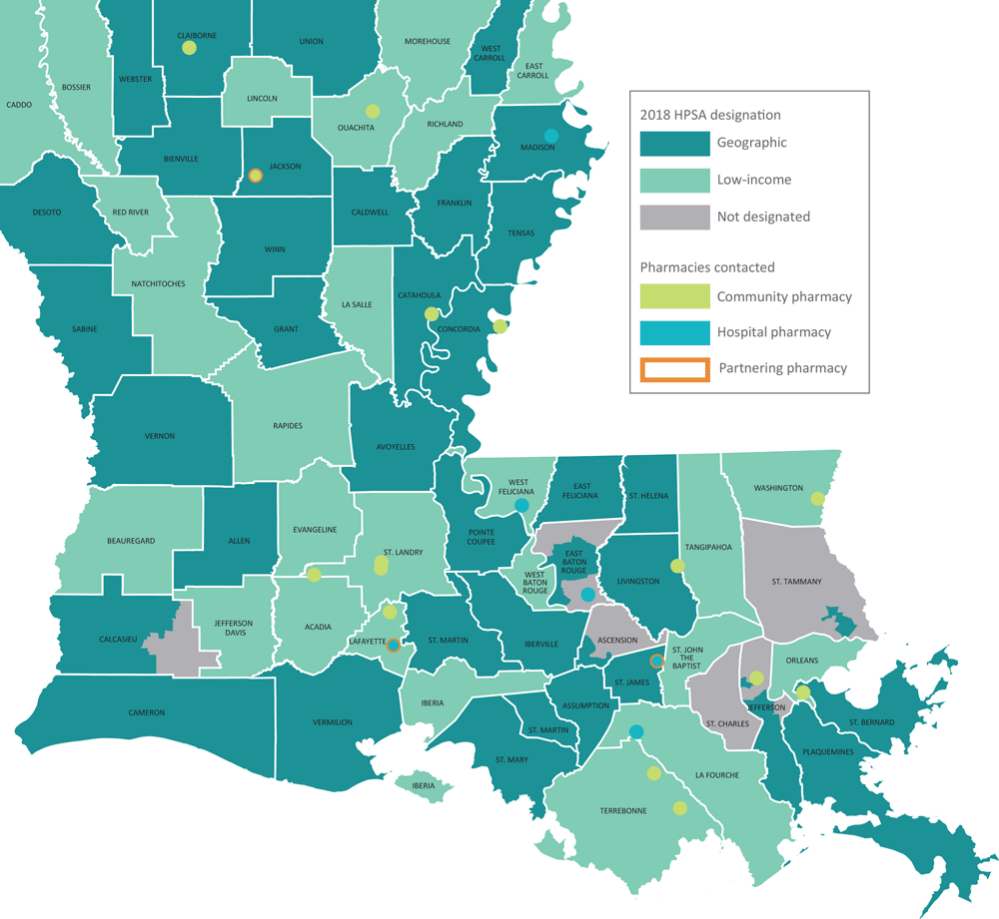
Location of geographic health professional shortage areas (HPSAs), low-income HPSAs, and 22 pharmacies that participated in a study on pharmacist–physician collaboration through collaborative drug therapy management (CDTM) agreements. A geographic HPSA designation is determined by the Health Resources and Services Administration (HRSA) as the ratio of the number of primary care providers to the number of people in a census tract, and a low-income HPSA designation is determined as the ratio of the number of primary care providers to the number of low-income people in a census tract (11).
- Allow eligible providers to enter into a contract as long as they can be “physically present as needed,” rather than at all times. This modification would bring Louisiana in line with most other states offering CDTM, which do not have such strict physical proximity requirements. These states demonstrate that requiring the physical presence of a provider at all times is not necessary for the viability of CDTM.
- If CDTM agreements are to become viable tools for pharmacists, the record-keeping burden must be reduced. Many states allow a CDTM agreement to define the scope of care, outlining what the pharmacist is authorized to do and for which diseases or conditions, rather than requiring the agreement to include the names of covered patients, as Louisiana does (7). This change would provide more flexibility for the collaborating providers to add new patients without having to update the agreement. Further, the CDTM could cover prospective patients, including those referred within a short time frame, such as in the example of the rural hospital described earlier. Removing the annual renewal requirement would also reduce the administrative burden of CDTM.

We believe any or all of these changes would increase the number of pharmacists participating in CDTMs in Louisiana. Pharmacists we have spoken with have demonstrated a strong interest in partnering with their physician counterparts, but the perceived and experienced barriers of CDTM have discouraged them from pursuing such an agreement. CDTMs are a powerful tool that can improve care for patients with chronic disease and empower our medical workforce to provide care at the top of their license. By removing these restrictions, we can maximize the agreements’ functionality and allow pharmacists to adopt a stronger role in chronic disease treatment.
